# Comparing Two Days of Dietary Intake in What We Eat in America (WWEIA), NHANES, 2013–2016

**DOI:** 10.3390/nu13082621

**Published:** 2021-07-29

**Authors:** Lois C. Steinfeldt, Carrie L. Martin, John C. Clemens, Alanna J. Moshfegh

**Affiliations:** Food Surveys Research Group, Beltsville Human Nutrition Research Center, Agricultural Research Service, USDA, Beltsville, MD 20705, USA; Lois.Steinfeldt@usda.gov (L.C.S.); John.Clemens@usda.gov (J.C.C.); Alanna.Moshfegh@usda.gov (A.J.M.)

**Keywords:** What We Eat in America, NHANES, automated multiple-pass method, dietary intake

## Abstract

The objective of this research is to compare the Day 1 and Day 2 dietary intakes of adults in What We Eat in America, National Health and Nutrition Examination Survey (WWEIA, NHANES) 2013–2016. Dietary recalls of males (*n* = 2599) and females (*n* = 2624) 20+ years who had both a Day 1 and Day 2 recall and reported their intake as usual on both days in WWEIA, NHANES 2013–2016 were examined. Mean (±SE) energy intake for males was 2425 ± 26 kcal for Day 1 and 2334 ± 32 kcal for Day 2 (*p* = 0.004). For females, 1832 ± 18 kcal and 1775 ± 26 kcal were reported for Day 1 and 2, respectively (*p* = 0.020). There were no significant differences between energy intake on Day 1 and Day 2 within males and females by ten-year age groups. Comparing 20 year age groups for males and females by race/ethnicity (non-Hispanic White, non-Hispanic Black, non-Hispanic Asian, and Hispanic) and income (<131%, 131–350%, and >350% of poverty level) also showed no significant differences in energy intake between Day 1 and Day 2. Mean energy intake of adults was not statistically different between the two days of recall by sex, race/ethnicity or income within selected age groups. Overall, the difference in energy intake was less than 4% for both males and females.

## 1. Introduction

Dietary intake is an important factor for health promotion and disease prevention. Foods and nutrients have been linked as both contributing to and preventing chronic diseases such as type 2 diabetes, cardiovascular disease, and certain cancers [1,2]. Dietary intake can also affect energy levels, muscle and bone health, immune response, and mental health status [3]. What We Eat in America, National Health and Nutrition Examination Survey (WWEIA, NHANES) collects two 24 h dietary intakes on a representative sample of about 5000 individuals each year along with demographic, socioeconomic, medical, dental, laboratory, and other health related data [4].

One important influence on intake is the day of the week [5] which reflects many aspects of lifestyle including work and school schedules, physical activity, and social functions. In particular, intake of many foods and beverages, and thus energy intake and macronutrient distribution, tend to be different on weekends (Friday through Sunday) than on weekdays (Monday through Thursday). A study of WWEIA, NHANES 2003–2012 found weekend consumption was associated with increased calorie intake and lower quality diets [6]. In addition, weekend-weekday differences in diet have been found to be more pronounced in women than in men and in younger adults than in older adults [7]. Day-of-week effects can be minimized by both study design and analysis procedures.

The source of foods and beverages, for example, restaurants, grocery store, schools, etc., has also been identified as having an impact on both diet quality and intake [8]. Previous studies using WWEIA, NHANES data have indicated consumption from fast-food and full-service restaurants are associated with an increase in total daily energy intake [9,10]. The U.S. Department of Agriculture’s (USDA) Economic Research Service found that the energy contribution from food prepared away from home, which includes restaurants, doubled over the past four decades [11]. Identification of food source is an important detail to note during the collection of 24 h recalls.

Another influence on dietary intake occurs when individuals change their behavior due to awareness that their behavior is being or will be measured. This change in behavior is called reactivity [12] and may include changes in eating behavior to reduce the foods consumed or to change what is consumed to meet a perceived standard for a healthy diet. The participant may also record the foods eaten in order to shorten the intake interview. When an individual changes their dietary intake, the resulting data may accurately represent what they ate that day but will not reflect how they usually eat.

Mean intake of foods/beverages and nutrients or other dietary components can be estimated using a single 24 h recall. However, collecting a 24 h recall for at least two non-consecutive days allows the application of statistical techniques to estimate usual dietary intake distributions for a population. For example, usual dietary intake is used to estimate the proportion of population groups meeting Dietary Guidelines for Americans recommendations [13] or Dietary Reference Intakes [14]. Two days of dietary intake in NHANES have also been used to generate within-person comparisons of eating behaviors such as weekday/weekend, eating at restaurants, alcoholic consumption, snacking, and eating breakfast [9,10,15,16,17]. However, there have been no studies examining the differences in energy intake between Day 1 and Day 2 by sex, age, race/ethnicity, and income.

The purpose of this study is to compare the Day 1 and Day 2 mean energy intakes of adults collected in WWEIA, NHANES 2013–2016 and to explore some of the factors which can create variation in mean daily energy intake.

## 2. Materials and Methods

### 2.1. Study Population

Dietary intake data from 2013–2014 and 2015–2016 WWIEA, NHANES [18,19] for adults aged 20 years and over were used in the analysis. For each two-year survey period, 24 h dietary intake recalls were collected using the USDA’s Automated Multiple Pass Method (AMPM) on nationally representative samples. USDA has conducted national surveys on the foods and beverages consumed by Americans using the 24 h recall method since 1965. In 2002, USDA developed and began using the Automated Multiple Pass Method (AMPM) [20] which is a validated, research-based, multiple-pass approach designed to encourage complete and accurate food recall and to reduce respondent burden [21,22]. The 5-step multiple-pass method used in AMPM leads the respondent through the 24 h period of the recall more than once using different approaches to assist the respondent in recalling foods and beverages without being repetitive [23]. AMPM provides a structured interview with standardized questions combined with unstructured opportunities for respondents to use their own individual strategies to remember and report foods and beverages.

The Day 1 dietary intake was collected in the Mobile Exam Center (MEC) and the Day 2 dietary intake was collected 3–10 days later during a scheduled phone interview. The Day 2 interview was not collected on the same day of the week as the Day 1. Additionally, if the Day 1 interview was collected on a weekend day (Friday, Saturday, or Sunday), the survey design called for the second day to be collected on a weekday (Monday–Thursday) and vice versa. At the end of each dietary interview, the participant was asked to characterize the amount of food they ate for that day as “much more than usual”, “usual”, or “much less than usual” [24,25]. All foods and beverages reported during the AMPM interviews were assigned food codes using USDA’s Food and Nutrient Database for Dietary Studies (FNDDS) 2013–2014 [26] and 2015–2016 [26]. The FNDDS converts foods and beverages consumed in WWEIA, NHANES into gram amounts and determines the corresponding nutrient values.

The NHANES participants were selected based on a national probability design [27]. To increase the number of participants for specific demographic groups, a multi-stage, unequal probability of selection design was implemented. In the 4 years for 2013–2016 there were a total of 10,064 individuals aged 20 and older. Of these 8672 individuals had 2 complete 24 h dietary intakes with no fasting days and were included in the analyses for recall collection on weekend day versus weekday, restaurant intake, and usual amount.

Day 1 or Day 2 was classified as a restaurant day if the source of at least one reported food or beverage item was from a restaurant. This classification has been used in previous research on restaurant days [9,10] and is used by USDA in the What We Eat In America, NHANES data tables [28]. In this analysis, restaurant included source of food coded as: “Restaurant with waiter/waitress”, “Bar/tavern/lounge”, “Restaurant no additional information”, “Cafeteria NOT in K-12 school”, “Restaurant fast food/pizza”, “Sport, recreation, or entertainment facility”, or “Street vendor, vending truck”. Additional information on all selections available for source of food can be found in the NHANES documentation [24,25].

During each dietary recall interview in AMPM, the participant is asked to describe the amount of food eaten that day as “More than Usual”, “Usual”, or “Less than Usual”. This is referred to in this paper as self-described intake. A total of 5223 individuals had 2 days of dietary intake where they reported the amount of food consumed as usual for both days and were included in the analysis for comparison of mean daily energy intakes on Day 1 versus Day 2.

### 2.2. Statistical Analysis

To produce national, representative estimates, the appropriate sample weights must be used [24,25]. Participants with both Day 1 and Day 2 dietary intake data had two-day dietary weights applied. Although attempts are made to schedule MEC exams which include the Day 1 dietary intakes throughout the week, proportionally more exams occur on weekend days than on weekdays. Because food intake can vary by weekdays and weekends [5,6,7], use of the MEC weights disproportionately represents intakes on weekends. The two-day dietary weights adjust for the additional non-response for the second recall and the proportion of weekend (Friday through Sunday) and weekday (Monday through Thursday) combinations of Day 1 and Day 2 recalls. These two-day dietary weights were applied in the analyses for restaurant intake, usual amounts, and comparison of mean energy intake on Day 1 compared to Day 2.

Analyses were conducted using SAS^®^ 9.4 (2012, SAS Institute Inc., Cary, NC, USA) and SAS-Callable SUDAAN version 11.0 (RTI International, Research Triangle Park, Durham, NC, USA) to adjust for survey design effects resulting from the NHANES complex, multistage probability sampling. Sample weights designed for dietary analysis were applied in all analysis to provide estimates of intake of nutrients reflective of the U.S. population during the survey timeframe. All comparisons were done using *t*-tests. Because of the large number of comparisons performed, results with *p* < 0.001 were considered statistically significant in all analyses.

## 3. Results

### 3.1. Sociodemographic Characteristics

Sociodemographic characteristics of the 5223 participants used in the comparison of energy intake between Day 1 and Day 2 by sex and age are provided in Table 1. Distributions of race/ethnicity were similar for both sexes with non-Hispanic (NH) Whites comprising the largest percentage among both males and females and NH Asians the smallest percentage. Distributions of race/ethnicity were also similar by age except for age 60+ years where the percentage of NH Asians was 6–8% versus 10–15% among the other age groups. For age 60+ years the percentage of NH Whites was 52–54% versus 40–43% among the other age groups.

Income levels reflect family income in terms of the Poverty Income Ratio (PIR) which is the ratio of family income to poverty expressed as a percentage [29]. The categories of income as percentage of poverty used for this analysis were <131%, 131–350% and >350%. Distributions of family income as PIR were similar across both sexes with those whose family income was at the middle level of 131–350% PIR comprising the largest percentage among both males and females. This was followed by those whose family income was >350% PIR and <131% PIR. Distributions of family income by age were generally similar.

### 3.2. Weekend/Weekday

Day 1 and Day 2 24 h dietary recalls, hereafter referred to as recalls, were conducted as part of the in-person MEC interview and scheduled phone interview, respectively. There were 8672 participants with 2 days of recalls where neither day was a fasting day. The number of days between the two recalls varied with a mean of 7 days. Approximately 90% of the Day 2 interviews were completed within the 3-to-10-day protocol established in the survey design. The Day 1 recall was collected on a weekend day for 62% of all participants and was the same percentage for both males and females. When investigated by age, both males and females 60 years and older had the smallest percentage of the Day 1 recalls collected on a weekend day at 50%, again with no difference between males and females. The percentage of Day 1 recalls collected on a weekend day for younger age groups ranged from 64–73%. When assessed by race/ethnicity, NH Asians and Hispanics had the highest percentage of the Day 1 recalls collected on a weekend day, 70% and 66%, respectively, versus 62% of NH Blacks and 57% of NH Whites. The percentage of Day 1 recalls collected on a weekend day was similar across all levels of income and ranged from 61–64%. A total of 73% of study participants had the recalls collected on both a weekend and weekday while 18% had 2 weekday recalls and 9% had 2 weekend day recalls [30]. All weekend/weekday percentages are unweighted.

### 3.3. Restaurant Intake

Table 2 provides the mean daily energy intake differences by restaurant reporting status, sex, and intake day. Overall, 54% of participants reported consumption of a restaurant food or beverage on Day 1 and 50% on Day 2. When assessed by sex, 55% of males reported consumption of a restaurant food or beverage on Day 1 and 53% on Day 2 compared to 52% and 48% of females on Day 1 and Day 2, respectively. Mean daily energy intake was significantly higher among consumers of restaurant foods and beverages versus non-consumers for both Day 1 and Day 2 overall as well as by sex (*p* < 0.001). The difference in energy intake for males was 324 and 235 calories on Day 1 and Day 2, respectively. The difference in energy intake for females was 190 and 239 calories on Day 1 and Day 2, respectively.

### 3.4. Self-Described Intake

Overall, 75% of participants characterized the amount of foods and beverages consumed as usual on Day 1 and 83% on Day 2. On Day 1, 9% reported amounts as more than usual and 16% reported less than usual. The percentages for Day 2 were 6% and 12%, respectively. In general, a larger percentage of males reported amounts as usual compared to females for both days. Older adults, NH Whites, NH Asians, and those with higher incomes also had higher percentages reporting usual amounts [30].

Table 3 provides the mean daily energy intakes by self-described intake, sex, and intake day. Differences in mean daily energy intake for males between those reporting their intake as more than usual and those reporting usual were 462 calories on Day 1 and 375 calories on Day 2. Differences between those reporting usual and less than usual were 298 calories for Day 1 and 344 calories for Day 2. All differences for males were significant (*p* < 0.001). Differences for females on Day 1 between those reporting more than usual and those reporting usual were 249 calories on Day 1 (*p* = 0.007) and 276 calories on Day 2 (*p* < 0.001). Differences between females reporting usual and less than usual were 166 calories on Day 1 and 417 calories on Day 2. Both of these differences were significant (*p* < 0.001).

### 3.5. Comparison of Day 1 and Day 2 Mean Energy Intake

Table 4 provides the mean daily energy intake by sex, age, and intake day. Only those participants with self-described intakes reported as usual on both days were included in this analysis. There were no significant differences in mean daily energy intake between days overall or by age groups among either males or females at the predetermined level of *p* < 0.001. Mean energy intake for all adults age 20 years and older was 2130 calories on Day 1 and 2056 calories on Day 2 with no significant difference. In the following results presented, a positive difference in energy intake corresponds to a higher energy intake on Day 1 than Day 2 and a negative difference in energy intake corresponds to a lower intake on Day 1 than Day 2. The difference in energy intake between Day 1 and Day 2 for the different age groups among males ranged from −37 kcal for age 60+ to 199 kcal for age 20–29 years, and among females ranged from −28 calories for age 60 plus to 135 kcal for age 30–39 years. These differences in energy intake between Day 1 and Day 2 correspond to 2–8% of Day 1 mean energy intake.

Table 5 provides the mean daily energy intake by sociodemographic characteristics, sex, age, and intake day. There were no significant differences in mean daily energy intake between days by race/ethnicity or income at the predetermined level of *p* < 0.001. The difference in energy intake between Day 1 and Day 2 for the different ethnicities among males ranged from −229 kcal for NH Asians age 60 plus to 544 kcal for NH Blacks age 20–39 years, and among females ranged from −35 kcal for NH Whites age 60 plus to 191 kcal for NH Asians age 20–39 years. These differences in energy intake between Day 1 and Day 2 correspond to 2–20% of Day 1 mean energy intake. The difference in energy intake between Day 1 and Day 2 for the different income levels among males ranged from −36 kcal for 131–350% PIR age 40–59 years to 265 kcal for <131% PIR age 40–59 years, and among females ranged from −61 kcal for 131–350% PIR age 60 plus to 156 kcal for <131% PIR age 20–39 years. These differences in energy intake between Day 1 and Day 2 correspond to 4–11% of Day 1 mean energy intake.

There were no significant differences in mean daily energy intake between Day 1 and Day 2 weekend days for males or females overall. The same was true for weekdays. When comparing Day 1 and Day 2 for restaurant days and non-restaurant days separately, there were no significant differences in mean daily energy intake for males or females overall.

## 4. Discussion

Capturing dietary intake in a manner that minimizes bias and error is important for research on associations between diet and chronic disease as well as development of public health policy. The comparison of the Day 1 and Day 2 mean energy intakes of adults in WWEIA, NHANES 2013–2016 showed no significant differences by sex, race/ethnicity, or income within selected age groups. Overall, the difference in mean energy intake between Day 1 and Day 2 was less than 4% for both males and females.

These results demonstrate the success of the AMPM 24 h dietary recall data collection used in the WWEIA, NHANES protocol for dietary interviews, the training and skill of the interviewers administering the AMPM, and the effectiveness of the two-day dietary weights which adjust for weekend/weekday differences. This is achieved despite the variation in dietary intakes that is common within and between individuals.

The consistency between the 2 days also suggests that reactivity to the Day 1 interview caused minimal bias during data collection of the Day 2 interview. The mean timeframe of 7 days between the Day 1 and Day 2 dietary data collection appears sufficient to minimize behavior change or diet awareness due to the Day 1 dietary recall process. Because 89% of the Day 2 intakes were collected within 3–10 days, there were insufficient scheduling differences to examine the effect on energy intake by the length of time between the dietary interviews. It should be noted that, although the mean number of days between Day 1 and Day 2 is 7, no Day 2 interviews were conducted exactly 7 days apart, i.e., the same day of the week as the Day 1 interview as specified in the protocol.

The fact that dietary intakes differ based on collection occurring on a weekday versus weekend day has been previously identified as an important factor contributing to variation [6,7]. However, the protocol in WWEIA, NHANES was largely adhered to with 72% of participants having one of the two days on a weekend and the other on a weekday. In addition, the dietary sample weights applied in analysis adjust for cases where 2 weekdays or 2 weekend days were collected for a participant and for the uneven distribution by the day of the week. The higher percentage of younger adults interviewed on a weekend day rather than weekday for Day 1 suggests the possibility of scheduling issues among younger adults who may be more likely to be unavailable during weekday work hours. This should be considered when developing study protocols related to dietary data collection for this age group.

Another eating behavior that increases variation in the direction of higher energy intake is consumption of restaurant foods which increased energy intake significantly for both males and females and which also occurs more frequently on weekends. With 54% of participants reporting consuming at least one restaurant food on Day 1 and 50% on Day 2, this has the potential to substantially impact the consistency of energy intake between dietary intakes collected on different days. In this analysis, mean daily energy intake differences between restaurant and non-restaurant days ranged from 190 to 324 calories. A slightly higher percentage of restaurant days (30%) were excluded from the mean daily energy intake comparison analysis because the intake was reported as more or less than usual compared to non-restaurant days (28%) for Day 1. The percentages for Day 2 showed the same pattern although about 10% lower (restaurant days: 21% vs. non-restaurant days 18%). Identification of the source of foods and beverages during dietary data collection, i.e., grocery store, restaurant, etc., is an additional variable which should be considered in the analyses and may help to explain some of the variation seen in dietary intakes.

Overall, participants characterized three-quarters of their intake days as usual in terms of the total amount of food consumed. Although the participant’s self-described intake as usual, more than usual, or less than usual is a subjective measurement, in this analysis the mean daily energy intakes were significantly higher for days reported as more than usual and significantly lower for those reported as less than usual. This suggests that participants were able to characterize their intake amount, at least generally.

Common limitations observed with dietary data collection using 24 h dietary recalls have been identified and evaluated regarding the dietary data collection for WWEIA, NHANES specifically [4,12]. It should also be noted that although this analysis used 2 cycles of WWEIA, NHANES, each cycle is a different group of participants who although nationally representative might produce different results.

An important strength of this analysis is the use of 2 cycles or 4 years of WWEIA, NHANES which is a nationally representative survey. These nationally representative estimates result from the NHANES sample design and the application of dietary sample weights, which include an adjustment for day of the week as well as adjustments for the basic probability of selection and for nonresponse. In addition, the detailed and carefully controlled protocols for collecting dietary intakes provide consistent and high-quality data.

## 5. Conclusions

Mean daily energy intake of adults was not statistically different between the two days of recall by sex, race/ethnicity or income within selected age groups. Overall, the difference in energy intake was less than 4% for both males and females. This indicates that the WWEIA, NHANES dietary intake protocols and statistical weighting results in successful collection of comparable intakes for Day 1 and Day 2.

## Figures and Tables

**Table 1:** Sociodemographic characteristics of U.S. adults by sex and age, What We Eat in America, National Health and Nutrition Examination Survey (WWEIA, NHANES) 2013–2016 ^1^.

Characteristic	Males			Females		
	Age 20–39 y ^2^ No. (%)	Age 40–59 y No. (%)	Age 60+ y No. (%)	Age 20–39 y No. (%)	Age 40–59 y No. (%)	Age 60+ y No. (%)
Race/Ethnicity						
Non-Hispanic White	325 (40)	353 (42)	493 (52)	338 (41)	386 (43)	485 (54)
Non-Hispanic Black	129 (16)	154 (18)	165 (18)	128 (15)	162 (18)	116 (13)
Non-Hispanic Asian	122 (15)	117 (14)	58 (6)	100 (12)	93 (10)	73 (8)
Hispanic	186 (23)	196 (23)	211 (22)	223 (27)	230 (26)	200 (22)
Non-Hispanic Other	46 (6)	30 (3)	14 (2)	41 (5)	31 (3)	18 (2)
Family Income asPIR ^3^						
<131%	226 (28)	198 (23)	234 (25)	288 (35)	223 (25)	236 (26)
131–350%	298 (37)	259 (30)	357 (38)	279 (34)	287 (32)	335 (38)
>350%	222 (27)	328 (39)	277 (29)	212 (25)	319 (35)	225 (25)
Income N/A	62 (8)	65 (8)	73 (8)	51 (6)	72 (8)	96 (11)

^1^ 5223 individuals with 2 days of intake with both days reported as usual amounts. ^2^ Years. ^3^ PIR (poverty income ratio) is the ratio of family income to poverty, expressed as a percentage.

**Table 2:** Mean daily energy intake differences by restaurant reporting status, sex, and intake day for U.S. adults age 20+, WWEIA, NHANES 2013–2016 ^1^.

	% Reporting	Restaurant	No Restaurant	Diff	*p*-Value
		Mean Daily Energy Intake (SE)			
Males					
Day 1	55	2589 (38)	2265 (32)	324	<0.001
Day 2	53	2421 (47)	2187 (27)	235	<0.001
Females					
Day 1	52	1918 (18)	1728 (20)	190	<0.001
Day 2	48	1864 (27)	1625 (26)	239	<0.001

^1^ Dietary two-day sample weights applied in analysis.

**Table 3:** Mean daily energy intakes by self-described intake, sex, and intake day for U.S. adults age 20+, WWEIA, NHANES 2013–2016 ^1^.

	% Reporting Usual	Self-Described Intake			Difference More vs. Usual	*p*-Value	Difference Less vs. Usual	*p*-Value
		More than Usual	Usual	Less than Usual				
		Mean Daily Energy Intake (SE)						
Males								
Day 1	77	2917 (111)	2455 (24)	2156 (54)	462	<0.001	298	<0.001
Day 2	83	2704 (68)	2329 (28)	1985 (54)	375	<0.001	344	<0.001
Females								
Day 1	73	2084 (78)	1835 (15)	1669 (35)	249	0.007	166	<0.001
Day 2	81	2049 (62)	1772 (22)	1356 (37)	276	<0.001	417	<0.001

^1^ Dietary two-day sample weights applied in analysis.

**Table 4:** Mean daily energy intake by sex, age, and intake day for U.S. adults age 20+, WWEIA, NHANES 2013–2016 ^1^.

Age (y)	Males (*n* = 2599)		Diff	% Day 1 Intake	Females (*n* = 2624)		Diff	% Day 1 Intake
	Day 1	Day 2			Day 1	Day 2		
	Mean Daily Energy Intake (SE)				Mean Daily Energy Intake (SE)			
20+	2425 (26)	2334 (32)	91	3.8	1832 (18)	1775 (26)	57	3.1
20–29	2561 (57)	2362 (68)	199	7.8	1970 (52)	1868 (52)	102	5.2
30–39	2625 (76)	2562 (80)	63	2.4	1942 (39)	1807 (39)	135	7.0
40–49	2445 (72)	2302 (92)	143	5.8	1844 (52)	1758 (44)	86	4.7
50–59	2463 (52)	2314 (53)	150	6.1	1809 (40)	1758 (41)	50	2.8
60+	2175 (37)	2212 (39)	−37	1.7	1695 (38)	1723 (45)	−28	1.7

^1^ Dietary two-day sample weights applied in analysis.

**Table 5:** Mean daily energy intake by sociodemographic characteristics, sex, age, and intake day for U.S. adults age 20+, WWEIA, NHANES 2013–2016 ^1^.

Race/Ethnicity, Income, and Age (y)	Males		Diff	Females		Diff
	Day 1	Day 2		Day 1	Day 2	
	Mean Daily Energy Intake (SE)			Mean Daily Energy Intake (SE)		
Race/ethnicity Non-Hispanic White						
20–39	2547 (60)	2525 (83)	23	1973 (53)	1900 (50)	73
40–59	2502 (64)	2337 (77)	165	1831 (41)	1759 (43)	72
60+	2210 (43)	2252 (46)	−42	1705 (44)	1740 (55)	−35
Non-Hispanic Black						
20–39	2751 (160)	2207 (103)	544	1965 (89)	1777 (83)	188
40–59	2505 (117)	2118 (76)	387	1797 (57)	1774 (59)	23
60+	1993 (68)	1965 (81)	28	1677 (86)	1659 (127)	18
Non-Hispanic Asian						
20–39	2134 (94)	1940 (88)	194	1850 (75)	1659 (93)	191
40–59	2184 (81)	2194 (109)	−10	1764 (91)	1783 (83)	−20
60+	1881 ^3^ (86)	2110 ^3^ (286)	−229	1542 ^3^ (103)	1562 ^3^ (65)	−20
Hispanic						
20–39	2796 (104)	2618 (93)	178	1938 (87)	1763 (58)	175
40–59	2300 (77)	2195 (82)	104	1832 (46)	1733 (42)	99
60+	1975 (92)	1931 (60)	45	1694 (62)	1637 (81)	57
Income ^2^ <131% PIR						
20–39	2706 (122)	2465 (133)	241	1920 (45)	1763 (59)	156
40–59	2452 (101)	2187 (78)	265	1683 (85)	1699 (56)	−17
60+	2265 (66)	2081 (86)	184	1571 (39)	1566 (45)	6
131–350% PIR						
20–39	2689 (61)	2524 (74)	165	2005 (53)	1900 (60)	105
40–59	2450 (92)	2487 (124)	−36	1844 (44)	1757 (48)	87
60+	2139 (61)	2156 (65)	−17	1732 (46)	1793 (71)	−61
>350% PIR						
20–39	2413 (72)	2365 (83)	48	1978 (87)	1849 (63)	129
40–59	2488 (61)	2281 (69)	207	1880 (42)	1794 (41)	86
60+	2247 (61)	2335 (77)	−88	1742 (67)	1747 (79)	−5

^1^ Dietary two-day sample weights applied in analysis. ^2^ Income as PIR (poverty income ratio) is the ratio of family income to poverty, expressed as a percentage. ^3^ Indicates an estimate that may be less statistically reliable due to small sample size.

## Data Availability

All data is publicly available at web sites referenced in [19,20,22,23].
